# Local stressors mask the effects of warming in freshwater ecosystems

**DOI:** 10.1111/ele.14108

**Published:** 2022-09-25

**Authors:** Olivia F. Morris, Charlie J. G. Loewen, Guy Woodward, Ralf B. Schäfer, Jeremy J. Piggott, Rolf D. Vinebrooke, Michelle C. Jackson

**Affiliations:** ^1^ Georgina Mace Centre for the Living Planet Department of Life Sciences Silwood Park Campus Imperial College London Berkshire UK; ^2^ Department of Ecology and Evolutionary Biology University of Toronto Toronto Canada; ^3^ Institute for Environmental Sciences University Koblenz‐Landau Landau in der Pfalz Germany; ^4^ Department of Zoology Trinity College Dublin The University of Dublin Dublin Ireland; ^5^ Department of Biological Sciences University of Alberta Alberta Canada; ^6^ Department of Biology University of Oxford Oxford UK

**Keywords:** antagonisms, aquatic ecology, dominance null model, global change, multiple stressors, stressor interactions

## Abstract

Climate warming is a ubiquitous stressor in freshwater ecosystems, yet its interactive effects with other stressors are poorly understood. We address this knowledge gap by testing the ability of three contrasting null models to predict the joint impacts of warming and a range of other aquatic stressors using a new database of 296 experimental combinations. Despite concerns that stressors will interact to cause synergisms, we found that net impacts were usually best explained by the effect of the stronger stressor alone (the *dominance* null model), especially if this stressor was a local disturbance associated with human land use. Prediction accuracy depended on stressor identity and how asymmetric stressors were in the magnitude of their effects. These findings suggest we can effectively predict the impacts of multiple stressors by focusing on the stronger stressor, as habitat alteration, nutrients and contamination often override the biological consequences of higher temperatures in freshwater ecosystems.

## Graphical abstract

Climate warming is a ubiquitous stressor in freshwater ecosystems, yet its interactive effects with other stressors are poorly understood. Using a meta‐analysis, here we show that the net impacts of stressor pairs can be explained by the effect of the stronger stressor alone (the dominance null model), especially if it was associated with human land use.

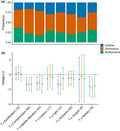

## INTRODUCTION

Our planet is rapidly warming with at least 1.5°C increase projected by 2052 (IPCC, 2021). Despite this, we still know very little about how ecosystems will respond to warming and even less about potential interactions with other stressors (Jackson et al., 2016; Paine et al., 1998; Sala et al., 2000). Several studies have quantified the net impacts of different stressor combinations, and there have been increasing attempts to synthesise this information (e.g. Ban et al., 2014; Bancroft et al., 2008; Crain et al., 2008; Darling & Côté, 2008; França et al., 2020; Jackson et al., 2016), yet there lacks a thorough assessment of climate warming interactions and the predictive power of contrasting null models. Past studies have been limited by the availability of data documenting responses to higher temperatures in different contexts (e.g. stressor combinations and levels of biological organisation) and most have only considered how the effects of paired stressors deviate from a single null model (e.g. Ban et al., 2014; Crain et al., 2008; Jackson et al., 2016; Przeslawski et al., 2015). We integrate recent evidence of multiple stressor interactions involving warming in a new formal meta‐analysis with a substantially increased sample size and statistical power to test the cumulative effects of multiple stressors on freshwater ecosystems and generate new insights into predicting the impacts of climate warming.

Multiple stressor studies often focus on pairs of stressors and assume the parsimonious *additive* model, whereby the combined effect of stressors is simply the sum of their independent effects. Deviation from this additive expectation is broadly categorised as antagonistic (less than *additive*) or synergistic (greater than *additive*). While antagonism and synergy can be defined relative to any null expectation (e.g. *multiplicative*; Folt et al., 1999) we use these terms in reference to the *additive* model for simplicity. The drivers of such non‐additivity are varied and often poorly understood, but one potential factor is the degree of asymmetry that exists between individual stressor effects (Darling et al., 2010; Sala et al., 2000). Degree of asymmetry refers to the imbalance or difference in stressor effects when they occur independently, whereby prevailing stressors with relatively strong impacts may suppress or negate weaker stressors. Several meta‐analyses suggest stressor asymmetry is important given the apparent prevalence of antagonistic effects (e.g. Cote et al. 2016; Jackson et al., 2016; Piggott et al. 2015; Tekin et al., 2020). Given the frequency with which stressors interact, a mechanistic understanding is needed if we are to predict future impacts in a rapidly changing—and warming—world (De Laender, 2018; MacLennan & Vinebrooke, 2021; Schäfer & Piggott, 2018). Instead of assuming additivity, it is increasingly argued that researchers should base predictions on null models selected using an established framework (e.g. Schäfer & Piggott, 2018), so that interactions can be anticipated a priori rather than simply dismissed as ‘ecological surprises’ (Orr et al., 2020).

**FIGURE 1:**
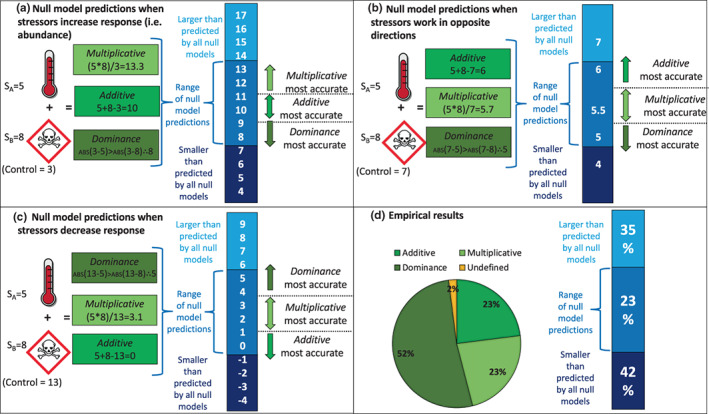
Examples of predicted responses from the *multiplicative*, *additive* and *dominance* models when stressor pairs (S_A_ = stressor a, S_B_ = stressor B) have positive (a), contrasting (b) or negative (c) effects relative to the control, which varies in the different scenarios (ABS = absolute). Responses include plant or animal survival, growth/size, condition, biomass, abundance, diversity and decomposition measured from experimental treatments. An overview of our empirical results (d) shows the best performing null models across all (296) responses and the percentage of observations that fell within or beyond the range predicted by all three null models. Undefined responses are those where multiple null models were indistinguishable in their performance.

Alternative null models include those commonly referred to as *‘multiplicative’* (which sums the proportional effects of stressors) and ‘*dominance’* (which takes the individual effect of the most impactful stressor; Folt et al., 1999; Figure 1). We might expect the *additive* model to yield the most accurate predictions when warming is paired with a mechanistically distinct stressor, such as habitat alteration (Srivastava & Vellend, 2005). This is because negatively correlated species responses (i.e. where those tolerant of one stressor are sensitive to the other; Vinebrooke et al., 2004) are more likely when stressors have different modes of action. On the other hand, the *multiplicative* model is expected to provide better predictions when an interaction between warming and another stressor (e.g. acidification) amplifies their individual effects (e.g. Davis et al., 2010; Schindler et al., 1996). Alternatively, a *multiplicative* interaction can dampen impacts when stressor responses are moderately correlated (i.e., several impacted species are sensitive to both stressors, decreased response in Figure 1c). Whether the *multiplicative* model predicts amplified or dampened responses depends on whether stressors increase or decrease responses (Figure 1a,c). However, the smallest impacts are predicted by the *dominance* model, where the effect of the strongest stressor overrides that of other stressors. Such redundant impacts may occur when individual effect sizes are highly asymmetric or strongly positively correlated because of similar modes of action (e.g. size‐selective effects of higher temperatures and invasive predators; Loewen et al., 2020). The *dominance* model is also expected to perform best at higher levels of organisation (i.e. communities vs populations) where tolerant organisms can compensate (i.e. provide biological insurance; Naeem & Li 1997) for more sensitive taxa (Jackson et al., 2016; Thompson et al., 2018a).

We examine the accuracy of null models to predict warming interactions in freshwater ecosystems because they are integrators of the cumulative impacts of climate change and other co‐occurring stressors (e.g. invasion, pollution, habitat alteration and eutrophication; Sala et al., 2000; WWF., 2020) across their catchments (e.g. Williamson et al., 2009). Most freshwater organisms are ectothermic, making them intrinsically sensitive to temperature change (Gunderson & Stillman, 2015). While thermal optima have been identified for an increasing number of aquatic species (e.g. Hasnain et al., 2018), there is lacking a general framework for predicting context dependencies in temperature response, including amplification or moderation by co‐occurring stressors (Schäfer & Piggott, 2018). Freshwater organisms are also frequently used in experiments to quantify multiple stressor effects and this large sample size provides a valuable model system for formal meta‐analysis integrating the effects of stressors and evaluating theoretical predictions. We applied the three null models outlined above (*additive*, *multiplicative* and *dominance*) to compare predicted and observed responses from our database of freshwater stressors paired with environmental warming. Following from Schäfer and Piggott's (2018) null model framework, we tested three main hypotheses (H):

- H1—Overall: The *dominance* model will be most successful when high degree of asymmetry exists among the effects of individual stressors (i.e. driving antagonistic interactions). Also, the *dominance* model will become increasingly accurate as asymmetry increases.
- H2—Stressor identity: The combined effect of mechanistically dissimilar stressors (i.e. biological responses are negatively correlated) will be best predicted by the *additive* model, while the *dominance* model will perform best when stressor effects are mechanistically redundant of the each other (i.e. responses are positively correlated). The *multiplicative* model is expected to provide the most accurate predictions when stressors alter the effects of one another (especially when effects are amplified as in synergistic interactions).
- H3—Organisational level: The *dominance* model will perform best at the community level (due to positive species interactions and compensatory dynamics), whereas the *additive* model should provide better predictions at the organism and population levels.

## METHODS

### Data collection

We extracted data from 71 published papers presenting responses from fully factorial two‐stressor experiments. We selected all studies that included warming as one of two simultaneous stressors, defining stressor as any natural or anthropogenic environmental factor that causes a quantifiable positive or negative biological response (Côté et al., 2016). To do this, we extracted all experimental responses that included a temperature treatment from a prior exhaustive literature search of paired freshwater stressors (up to the year 2013) by Jackson et al. (2016), which obtained 119 responses from 41 papers. We then more than doubled the size of this dataset by searching for updates to the literature following the same methods to supplement with new evidence published between 2013 and 2019 (see Supplementary Information 1 in Jackson et al., 2016 for further details of data search and selection criteria). Here, we searched the Web of Science database for papers published up to May 2019 with the key word combinations (‘multiple’ or ‘stressor’ or ‘factorial’ or ‘experiment’) and (‘temperature’ or ‘warming’) and (‘freshwater’ or ‘river’ or ‘stream’ or ‘lake’). In total, 3000 additional papers were obtained and screened for use in our study, from which a further 177 responses were retained from 30 new papers.

Once appropriate studies describing temperature interactions were identified, we collected data directly from text, tables, supplementary information or associated data depositories. If data were not accessible via these methods, we contacted corresponding authors for access. Finally, we used PlotDigitizer (plotdigitizer.sourceforge.net) to extract data directly from published figures for the few cases in which authors did not respond. We obtained the mean response, standard deviation and sample sizes for each treatment (stressor A; stressor B; stressors A and B and control with no stressor), along with several potentially relevant covariates and metadata, for each response meeting our search criteria. Reported stressors paired with temperature are acidification, contamination, change in food quantity, habitat alteration, biological invasion, change in light quality, change in nutrient levels and salinity. Responses included measures of plant or animal abundance, biomass, condition, diversity, growth/size and survival, as well as decomposition rates. Measures of proportional mortality were converted into survival (e.g. by subtracting from one) to make the metrics comparable. In cases where multiple responses (e.g. different organism groups) were reported in a single paper, each response was retained (see statistical analysis section below for handling of multiple responses in meta‐analytic models).

### Null model predictions

We calculated the predicted combined response to stressors for each observation using three contrasting null models initially proposed by Folt et al. (1999): the *simple additive, simple multiplicative* (also known as *Bliss independence* or *effect addition*) and *dominance* (also known as *simple comparative*) models. Alternative, equivalent formulations are used when model inputs are effects (i.e. where stressor‐driven change is compared to a control reference state; see Supporting Information 1 for further details). Null models predict the joint impact of warming and another stressor based on individual responses relative to controls when the stressors act independently, as follows:

#### *Simple additive* model

The response to stressor A (*S*_*A*_) added to the response to stressor B (*S*_*B*_) minus the control (C; to calculate effect). Since response variables such as abundance, biomass or diversity cannot be less than zero (i.e. there is no such thing as negative abundance), negative additive predictions in these cases were replaced with zero.

(1) $$S_{A}+S_{B}-C$$

#### Simple multiplicative model

*S*_*A*_ multiplied by *S*_*B*_, relative to the control

(2) $$\frac{S_{A}S_{B}}{C}\;$$

#### *Dominance* model

If the absolute difference between *S*_*A*_ and the control is greater than the absolute difference between *S*_*B*_ and the control, then the dominance null is equal to *S*_*A*_, otherwise it is equal to *S*_*B*_.

(3) $$\left\{\begin{array}{c} S_{A},\;\;\left|S_{A}-C\right|>\left|S_{B}-C\right|\\ \;S_{B},\;\;\;\;\;\;\;\;\;\;\;\;\;\;\;\;\;\;\;otherwise \end{array}\right.$$

### Statistical analysis

#### Response frequencies

For each observation (*n* = 296), we compared the empirical response to each of the three null model predictions. To evaluate model performance, we first determined if observed responses fell within the ranges predicted across all three models combined (i.e. between the lowest and highest null model predictions; Figure 1). We then determined which model was most accurate for each individual response and summarised comparisons across pre‐defined stressor type, organisational level (individual, population, community) and asymmetric stressor categorical groupings. Asymmetry was defined as one stressor causing an effect >50% larger than the second (compared to the control) when each was independent. We calculated degree of asymmetry as:

$$\;\left|\left(\frac{\left|S_{A}‐C\right|}{C}\right)\times 100-\left(\frac{\left|S_{B}‐C\right|}{C}\right)\times 100\;\right|$$

Additional comparisons across organism groups and response metrics are presented to provide further insights in the Supporting Information. Groupings with fewer than eight responses (*n* < 8) were excluded from analyses following Jackson et al. (2016).

#### Mean effect sizes

We used the *metafor* package in R (function ‘rma.mv’; Viechtbauer, 2010) to estimate mean interaction effect sizes and evaluate the accuracy of null model predictions across studies from weighted meta‐analyses. We used Hedges' *d* (Hedges, 1981) as a standardised mean difference (with bias‐correction for small sample sizes) between each model prediction and the observed (empirical) response for different categories of stressor pairs and response types. The directions of effect sizes were inverted (making negative effect sizes positive and vice versa) when predicted effects were negative relative to the control. By inverting negative responses, we were able to compare the strength of effect sizes on an absolute scale irrespective of direction (negative or positive; following Jackson et al., 2016). For example, negative effect sizes indicate smaller than predicted observed effects occurring when either positive responses are less positive than predicted or negative responses are less negative than predicted. We used multilevel (mixed‐effects) linear models treating categorical moderators (same groups as in our response frequency analysis) as fixed effects and ‘ID’ nested within ‘Study’ as random effects to allow true effect sizes to vary across observations and account for nonindependence of those from the same studies. Tests of residual heterogeneity (QE) and Wald‐type omnibus tests of moderators (QM) were used to assess the significance of unexplained variation and importance of categorical moderators for each model (see Supporting Information 2 for further details). Hedges' *d* was calculated as

(1) $$d=J\frac{X_{o}-X_{p}}{s},$$

with $X_{o}$ being the observed combined response to stressors A and B, $X_{p}$ being the predicted combined response (from each of three null models), J being a weighting factor to correct for small sample bias and s being the pooled standard deviation, where J and s were calculated as

(2) $$J=1-\frac{3}{4\left(n_{o}+n_{p}-2\right)-1},$$

and

(3) $$\begin{array}{c} s=\sqrt{\frac{\left(n_{o}-1\right){\left(s_{o}\right)}^{2}+\left(n_{p}-1\right){\left(s_{p}\right)}^{2}}{\left(n_{o}+n_{p}\right)-2}}, \end{array}$$

with $n_{o}$ being the sample size of the observed response (i.e. the combined stressor treatment), $n_{p}$ being the sample size of the predicted response (assigned to the minimum sample size of the two single‐stressor treatments used to calculate the predicted response), $s_{o}$ being the standard deviation of the observed response and $s_{p}$ being the standard deviation of the predicted response calculated by pooling the standard deviations of the two single‐stressor treatments. Mean interaction effect sizes with an absolute effect size greater than 30 were excluded from quantitative analyses and regarded as outliers (*n* = 7 excluded). Finally, the variances used to provide inverse weights of Hedges' *d* effect sizes (Vd) were calculated by

(4) $$\mathrm{Vd}=\frac{n_{o}+n_{p}}{n_{o}n_{p}}+\frac{d^{2}}{2\left(n_{o}+n_{p}\right)}.$$

#### Degree of asymmetry

We used generalised linear regression (using the ‘glm’ function with a Gamma error distribution and log‐link function; R Core Team, 2021) to investigate the relationship between accuracy of null model predictions (absolute effect sizes) and the degree of stressor asymmetry. Separate models were fit for observations where temperature was the stronger prevailing stressor (with an effect >50% larger than the second stressor), and where all other stressors were prevailing. Given the log‐link, a small constant (0.000000001) was added to absolute effect sizes for the analysis of asymmetric stressors where temperature was prevailing, to avoid negative values.

## RESULTS

### Response frequencies

**FIGURE 2:**
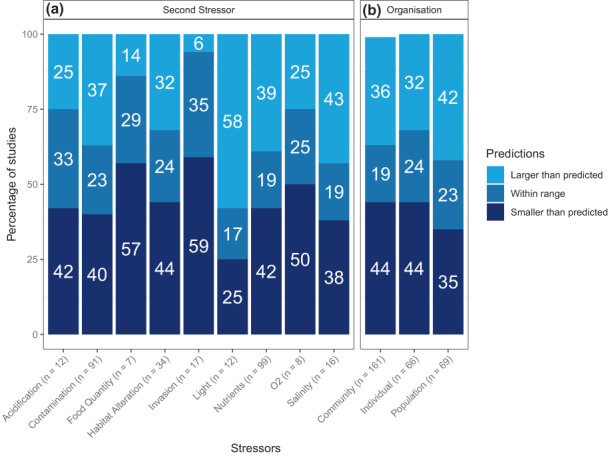
Percentage of observed responses to warming paired with another stressor that fall within or beyond the range predicted by all three null models grouped by stressor type (a) and organisation level (b) for the entire dataset (*n* = 296). Directions are on an absolute scale (e.g. observed responses larger than predicted were either more negative or more positive).

Ecological responses to climate warming paired with secondary stressors were outside the range predicted by all three null models in 77% of cases (Figure 2): 42% were less than predicted and 35% were greater (Figure 1c). The models were most likely to underestimate effects when temperature was paired with invasion or food quantity, while effects were overestimated when temperature was paired with light (Figure 2).

**FIGURE 3:**
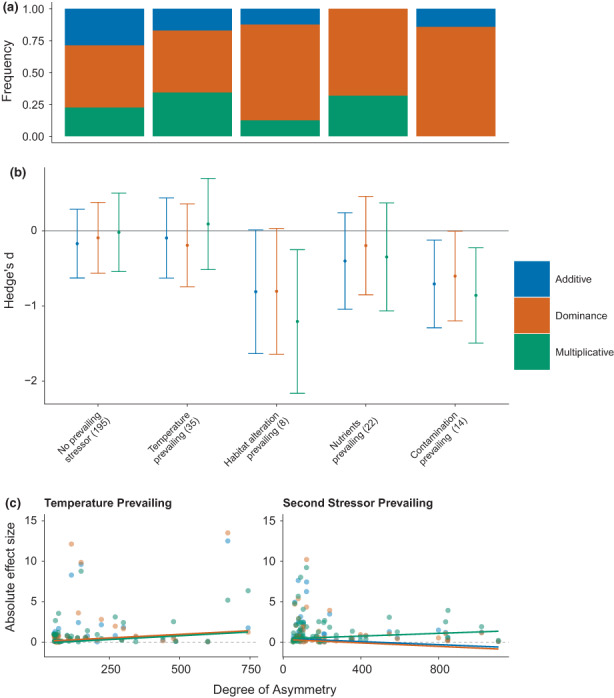
Asymmetric stressors. Frequency of responses best predicted by additive (blue), dominance (orange) and multiplicative (green) models (a) and their mean effect sizes (Hedges' *d*) showing standardised differences between observed effects and those predicted for each null model (with 95% confidence intervals; (b) across types of secondary stressors combined with temperature, when stressors were not strongly asymmetric or when either temperature, habitat alteration, nutrient levels or contamination had prevailing independent effects (effect >50% higher than the other). Mean effect sizes of zero indicate no difference between observed responses ($X_{o}$) and null model predictions ($X_{p}$). Mean observed effects are considered statistically indistinguishable from null model predictions when confidence intervals cross zero. Significant negative effect sizes indicate that observed responses were smaller (antagonistic; either less negative or less positive) than predicted. Although not seen here, significant positive effect sizes would indicate that observed responses were larger (more negative or more positive) than predicted. Panel (c) shows the absolute effect size for each null model with increasing asymmetry (% difference in independent effects) where either temperature or the second stressor are the prevailing independent stressor fitted with GLMs. Increasing (decreasing) absolute effect sizes indicate increasing (decreasing) deviation from null model predictions.

As hypothesised (H1), the *dominance* model performed best most often, outperforming the *additive* and *multiplicative* models in 52% of responses (152/296), while the other models most accurately predicted 23% of the observations each (Figure 1d). The *dominance* model performed the best in 62% of observations when the effect of one stressor was >50% larger than that of the second (i.e. asymmetric), (Figure 3a). The greater performance by the *dominance* null was especially apparent when habitat alteration, nutrients or contamination prevailed over warming (Figure 3a). The *multiplicative* model performed second best in instances of stressor asymmetry (25%) and the commonly applied *additive* model performed the worst (12%; Figure 3a). In these asymmetric scenarios, temperature was subordinate (i.e. the lesser stressor) in 56% of cases. In the 35 cases where temperature was the prevailing stressor, the *dominance* model was again the best, followed by the *additive* model (Figure 3a).

**FIGURE 4:**
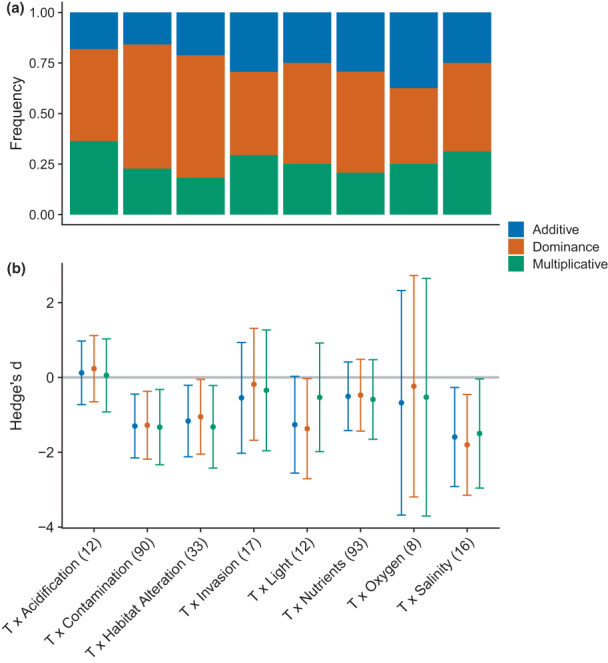
Stressor pairs. Frequency of responses best predicted by additive (blue), dominance (orange) and multiplicative (green) models (a) and mean effect sizes (Hedges' *d*) showing standardised differences between observed effects and those predicted for each null model (with 95% confidence intervals; (b) across types of secondary stressors combined with temperature, T. mean effect sizes of zero indicate no difference between observed responses ($X_{o}$) and null model predictions ($X_{p}$). Mean observed effects are considered statistically indistinguishable from null model predictions when confidence intervals cross zero. Significant negative effect sizes indicate that observed responses were smaller (either less negative or less positive) than null model predictions. Although not seen here, significant positive effect sizes would indicate that observed responses were larger (more negative or more positive) than predicted. Samples sizes may differ from Figure 1 because outliers were removed from mean effect calculations (see Methods).

**FIGURE 5:**
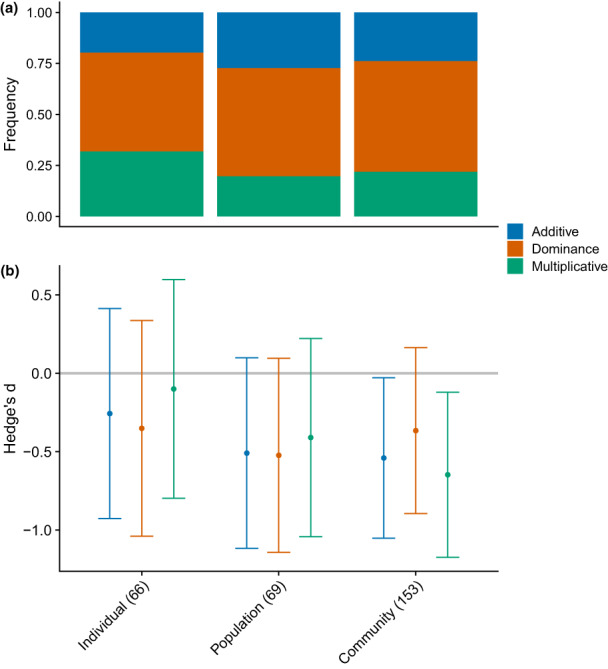
Biological organisation. Frequency of responses best predicted by additive (blue), dominance (orange) and multiplicative (green) models (a) and their mean effect sizes (Hedges' *d*) showing standardised differences between observed effects and those predicted for each null model (with 95% confidence intervals; (b) across levels of biological organisation. Mean effect sizes of zero indicate no difference between observed responses ($X_{o}$) and null model predictions ($X_{p}$). Mean observed effects are considered statistically indistinguishable from null model predictions when confidence intervals cross zero. Significant negative effect sizes indicate that observed responses were smaller (either less negative or less positive) than null model predictions. Although not seen here, significant positive effect sizes would indicate that observed responses were larger (more negative or more positive) than predicted.

Classifying responses by stressor pairs revealed similar patterns, with the *dominance* model most frequently outperforming the others when temperature was combined with contamination, nutrients, habitat alteration or change in light quality (Figure 4a). The consistently superior performance of the *dominance* null across stressor pairs countered our second hypothesis (H2) that model performance should depend on stressor mode of action and redundancy of effects. However, the *dominance model* best captured community‐level responses, supporting H3 (Figure 5a).

### Mean effect sizes

Contrasting the distinct differences in null model performance for single observations, prediction accuracy was similar when we combined warming responses across stressor asymmetries, types and organisation level to calculate weighted mean effect sizes (Figures 3b, 4b and 5b). Findings were similar for different organism and response metric groups (see Supporting Information 2 Figures 4 and 5); however, the *dominance* null performed better for primary producers as well as for responses that measured animal biomass or abundance. These results show greater variation among responses within groups (i.e. uncaptured context‐dependencies) than between null models. The importance of other moderating factors was supported by significant residual heterogeneity observed in each test (Supporting Information 2 Table S1).

In most cases, the mean effects of temperature interactions were classified as null (i.e. no difference between observed and predicted), regardless of model choice (Figures 3b, 4b and 5b). However, there were some exceptions, such as when warming was paired with light (mean effects were null only for the *multiplicative* prediction; Figure 4b), offering limited support for our second hypothesis (H2) as interaction classification depending on the type of secondary stressor. We also found that certain stressor combinations were predicted more accurately than others irrespective of model choice (Supporting Information 2 Table S1). Here, the combined effects of warming with acidification, invasive species, nutrients and oxygen were better predicted than those with contamination, habitat alteration and salinity, thereby consistently indicating antagonism.

We found that the prediction accuracy of our models generally decreased at higher levels of biological organisation (from individuals to communities; although differences among groups were nonsignificant; Supporting Information 2 Table S1), and that the *dominance* model performed best at the community level (supporting H3; Figure 5b). The smaller mean effect sizes (closer to zero) for the *dominance* model when habitat alteration, nutrient levels or contamination were the independently prevailing stressor, also supported our hypothesis that it should be used to make predictions when stressors differ strongly in their magnitudes of effect (Figure 3b). Prediction accuracy varied significantly across asymmetric stressor pairs for the multiplicative model only (Supporting Information 2 Table S1); however, predictions were generally most accurate (irrespective of null) when either temperature or nutrients were the prevailing stressor, and least accurate when contamination or habitat alteration were.

### Degree of asymmetry

Absolute combined effect sizes generally decreased (indicating greater prediction accuracy) with increasing asymmetry when secondary stressors (i.e. not temperature) were prevailing (Figure 3c). In these cases, the *dominance* null performed best, offering partial support of our first hypothesis. The opposite occurred (reduced prediction accuracy with increasing asymmetry) when temperature was prevailing (Figure 3c).

## DISCUSSION

The current climate crisis necessitates improvements to our limited ability to predict warming impacts and their interactions with other stressors, especially within vulnerable freshwater systems (Dudgeon et al., 2006). New frameworks are needed to guide the application of null models to forecast consequences of different stressor scenarios and inform the effective allocation of conservation resources. Using our uniquely comprehensive database of 296 experimental observations, we found evidence in support of our hypothesis that the *dominance* model best predicts the combined effect of warming and other stressors. This suggests that the ‘worst’, or most impactful, stressor frequently masks the effects of the weaker one, and the importance of stressor asymmetry was particularly apparent when habitat alteration, nutrients or contamination were prevailing (i.e. exerting effects >50% larger than temperature). Therefore, local stressors often appear to mask the more regional effects of higher temperatures linked to climatic warming. The *dominance* null performed best overall and provided more accurate predictions when temperature was subordinate as the degree of stressor asymmetry increased. However, this was dependent on stressor identity as the *dominance* null provided poorer predictions when warming had a prevailing independent effect.

These findings show that the impacts of warming may be harder to detect in already altered or contaminated habitats, which is the rule rather than the exception across many of the planet's freshwaters. As we show that the cumulative effects of anthropogenic activities on freshwater ecosystems often reflect those of the prevailing stressor, managers may be faced with having to also mitigate other worsening stressors once they negated the dominant local stressor and its antagonistic influence (Brown et al., 2013; Côté et al., 2016). However, despite clear differences in null model performance in individual studies, the *additive*, *multiplicative* and *dominance* models were largely indistinguishable when predicting mean effects across all studies (i.e. global scales), with a few notable exceptions. The similarity of mean effect sizes for different null models shows generally greater variation within categorical groups (e.g. secondary stressors and how responses are measured) than between model predictions, emphasising the need for further theoretical development of context‐dependent outcomes of stressor interactions.

### Response frequencies

Overall, the observed effect of stressor pairs frequently fell outside the range predicted by commonly used null models. In 42% of cases, the observed effect was less than predicted by all null models, supporting the previously documented prevalence of antagonistic stressor interactions in freshwater ecosystems (Jackson et al., 2016; Lange et al., 2018; Tekin et al., 2020). This was particularly apparent when temperature was combined with invasion or change in food quantity, which might be related to the similarity of their effects (Orr et al., 2022). For example, invasive predators and warming have similar size‐selective impacts (favouring smaller species; e.g. Loewen et al., 2020). When two stressors have redundant modes of action, exposure to the second stressor often has no (or a reduced) effect (Vinebrooke et al., 2004). Alternatively, stressors might interact to mitigate one another's impacts, resulting in a combined response which is less than even the effect of just one stressor alone (Côté et al., 2016; Jackson et al., 2016). For instance, nutrient additions can offset the rising metabolic demands of warming (Feehan et al., 2018) resulting in a combined effect that is less than warming alone.

Importantly, the *dominance* model predicted the smallest combined impact of the three models we considered and, therefore, performed best for the 42% of cases where the observed effect was below the full range of null model predictions. In fact, the *dominance* model was most accurate overall and, in support of our third hypothesis, we found that community‐level responses were most frequently predicted by the *dominance* model. Here, species interactions (e.g. predator–prey) and their density dependence (Brooks & Crowe, 2018; Thompson et al., 2018a), along with variation across spatial scales (Birk et al., 2020), complicate identification of the direct effects of stressors at higher levels of biological organisation. This could be due to positive species interactions (e.g. mutualisms) or compensation by stress‐tolerant species reducing the combined impacts of stressors within communities (as per the biological insurance hypothesis; Naeem & Li 1997). While most experiments are conducted in closed systems lacking opportunities for colonisation from the outside, increases in productivity by tolerant species arriving from the regional species pool may further offset functional losses owing to the decline of sensitive taxa in natural systems (Loewen & Vinebrooke, 2016). Thus, the *dominance* null may provide the best predictions at the community level due to strong compensatory species dynamics (as well as mutualisms, facilitations and trophic interactions) that maintain the robustness of functional properties in stressed freshwater ecosystems (Jackson et al., 2016; Schindler, 1987).

### Mean effect sizes

Mean effect sizes were generally similar between null models. For instance, results for all three models indicated that contamination combines with temperature to generate antagonistic effects (i.e. less than predicted). These findings support those from our response frequency analysis, showing that the effects of warming are especially difficult to detect when they are subordinate to habitat alteration, nutrients, or contamination. Several recent studies indicate that habitat loss and land‐use changes (which influence contaminant and nutrient levels) are among the most impactful drivers of biodiversity loss (Sala et al., 2000; Leclère et al., 2020). However, where warming effects are currently overshadowed by habitat alteration or loss of water quality, it remains unclear how changes to the relative strengths of stressors (i.e. accelerating climate change) might alter ecological outcomes for fresh waters. More research is needed to understand the nonlinearities of warming impacts and specifically when other concurrent stressors mask the effects of higher temperatures.

### Theoretical considerations

By synthesising accumulated evidence across independent studies, our meta‐analytical approach provides a novel means of assessing the generality of climate impacts on freshwater biodiversity and ecosystem functioning. The difficulty in predicting variation in climate change impacts stems, in part, from our limited understanding of the nature of interactions among multiple ecological stressors, and several syntheses of experiments have suggested nonadditivity is the norm (e.g. Crain et al., 2008; Jackson et al., 2016; Lange et al., 2018). Based on the concept of co‐tolerance (Vinebrooke et al., 2004), theory predicts that resistance to multiple stressors depends on the extent to which tolerances are correlated (i.e. positive, negative or random) and the potential for tolerant individuals or populations to compensate for sensitive ones. However, consequences will depend on how responses are measured, and nonlethal endpoints (e.g. growth or productivity) are often more challenging to predict as differing sensitivity correlations may result in similar null expectations (Schäfer and Piggot 2018). Responses may also be nonlinear, and it can be difficult to predict biological thresholds. Thus, differing sensitivities of metrics to the combined effects of multiple stressors may have contributed to the considerable heterogeneity of responses observed within groups of secondary stressors and levels of biological organisation.

Despite recent gains in our understanding of how multiple stressors are likely to interact, knowledge of interaction type (antagonistic or synergistic) does not itself provide an estimate of cumulative stressor impacts. For instance, Tekin et al. (2020) showed how the classification of stressor interactions can depend on choice of analytical framework and scaling of responses, but a second, more practical challenge remains in abstracting this information to generate empirical forecasts of use to managers (Dey & Koops, 2021). For instance, individual‐ or population‐level responses can be aggregated to reveal community impacts (e.g. via the compositional model from Thompson et al., 2018b). While compositional models require detailed taxonomic information on entire communities that is frequently unavailable, the decreasing predictive accuracy observed at higher levels of biological organisation suggests potential benefits of such approaches. Other tools, including concentration addition and stressor addition models, have been developed in the field of ecotoxicology, but attempts to apply these null expectations more generally have been hampered by their requirements for information on underlying stressor‐response/effect relationships and sensitivity distributions (Loewe & Muischnek 1926; Liess et al., 2016). As such data are typically lacking in multiple stressor studies, our empirical contrasts focus on the simple *additive*, *multiplicative* and *dominance* models (which are based on independent stressor effects without regard for level of organisation or response type).

We show the limits of our current comprehension of how stressors interact, as residual, unexplained heterogeneity emphasises the need for further development of a mechanistic framework taking additional context‐dependencies into account (Boyd et al., 2018; Burgess et al., 2021). For example, the cumulative effects of stressors and type of interaction may depend on stressor magnitudes, duration order of exposure (MacLennan & Vinebrooke, 2021) and type of response (i.e. organismal group, or level of biological organisation; Jackson et al., 2021, Orr et al., 2022). Further research under different stressor scenarios will be needed to disentangle the variation in effect sizes revealed by our analysis. Stressor combinations with relatively poorer prediction (such as when warming interacts with contaminants, habitat alteration or salinity) should be priorities. Until further advances are made, our work supports the assumption that the worse stressor will frequently overshadow stressors with smaller impacts, although cases of weaker stressors mitigating more impactful stressors can still occur. This conclusion supports that of Dey and Koops (2021), who advocated a phenomenological approach for predicting multiple stressor impacts to organism survival on the basis of stressor magnitude.

## CONCLUSIONS

We present a new global synthesis of experimental evidence on the cumulative effects of anthropogenic changes to freshwater ecosystems under climate warming. Building on prior knowledge that stressor interactions are commonly nonadditive (Piggott et al. 2015; Jackson et al., 2016; Tekin et al., 2020), we provide a novel, empirical comparison of the accuracy of three contrasting null models to predict the effects of rising temperatures in the context of multiple co‐occurring stressors. We found considerable heterogeneity within secondary stressor and biological groups, but the *dominance* model performed best most often. This model predicts a smaller combined effect than either the *additive* or *multiplicative* model, except when combined effects are less than that of the strongest stressor (or in the opposite direction relative to the control). While challenging to anticipate, the *multiplicative* null often performs best in these unique reversal scenarios.

We discovered that responses were similar irrespective of whether warming was the independently prevailing or subordinate stressor, with *dominance* an especially strong predictor for cases of stressor asymmetry. The accuracy of the *dominance* null increased with greater asymmetry between stressors, but only when temperature was subordinate. Climate warming impacts are thus more likely to be overshadowed by other stressors, rather than to amplify them. We urge caution when scaling these results up to the real world as short‐duration experiments may not always capture the full range of biological effects experienced by natural systems over longer terms, and we emphasise that responses were highly variable. However, our findings suggest that future impacts of climate warming may be less severe in previously degraded aquatic ecosystems.

## AUTHOR CONTRIBUTIONS

MCJ designed the study. OFM, CJGL and MCJ compiled the data. OFM and CJGL conducted the analysis and co‐wrote the first draft. All other authors contributed substantially to revisions.

### PEER REVIEW

The peer review history for this article is available at https://publons.com/publon/10.1111/ele.14108.

### OPEN RESEARCH BADGES

This article has earned an Open Data badge for making publicly available the digitally‐shareable data necessary to reproduce the reported results. The data is available at: https://doi.org/10.6084/m9.figshare.c.6156762.v1.

## Supporting information


Data S1

## Data Availability

All data and R code used in this paper can be found here: https://doi.org/10.6084/m9.figshare.c.6156762.v1
