# Supplementary material for: Local stressors mask the effects of warming in freshwater ecosystems
Source: Ecol Lett. 2022 Sep 25;25(11):2540–51. doi: 10.1111/ele.14108 (PMC9826496; doi:10.1111/ele.14108)
Supplement: Supplementary file 1 — Data S1 [file ELE-25-2540-s001.zip › ele14108-sup-0001-supplementary.docx]

**Local stressors mask the effects of warming in freshwater ecosystems**

Olivia F Morris^†^, Charlie JG Loewen^†^, Guy Woodward, Ralf B. Schäfer, Jeremy J. Piggott, Rolf D Vinebrooke and Michelle C Jackson*

^†^ denotes equal author contributions

* denotes corresponding author ([michelle.jackson@zoo.ox.ac.uk](mailto:michelle.jackson@zoo.ox.ac.uk))

**Supporting Information 1**

**Equivalent null model formations**

Our simple null models use the mean responses (S_A_, S_B_, and C) from experimental treatments as inputs. Equivalent formulations (from Schäfer & Piggot 2018) using effects as inputs are:

*f*_AB_(SI_A_, SI_B_) = *f*_A_(SI_A_) + *f*_B_(SI_B_) for the additive null,

*f*_AB_(SI_A_, SI_B_) = *f*_A_(SI_A_) + *f*_B_(SI_B_) – *f*_A_(SI_A_) *f*_B_(SI_B_) for the multiplicative null (where effects are converted to proportions of control), and

*f*_AB_(SI_A_, SI_B_) = max(*f*_A_(SI_A_), *f*_B_(SI_B_)) for the dominance null,

where *f*_A_(SI_A_) is the effect of stressor A for intensity SI_A_ and a given stressor-effect relationship *f*_A_, *f*_B_(SI_B_) is the effect of stressor B for intensity SI_B_ and a given stressor-effect relationship *f*_B_, and *f*_AB_(SI_A_, SI_B_) is the effect of stressors A and B combined for intensities SI_A_ and SI_B_ given a stressor-effect relationship *f*_AB_.

To show equivalence, we use the illustrative example of negative responses in the main text (Figure 1c), where treatment responses are S_A_ =5, S_B_ =8, and C = 13, and stressor effects (relative to the control) are therefore *f*_A_(SI_A_) = 8 (proportionally 0.385 or 38.5% remaining) and *f*_B_(SI_B_) = 5 (proportionally 0.615 or 61.5% remaining). Here, the additive effect *f*_AB_(SI_A_, SI_B_) is 5 + 8 = 13, implying a null expectation of 0 units remaining (C - *f*_AB_(SI_A_, SI_B_)). The multiplicative effect *f*_AB_(SI_A_, SI_B_) is 0.385 + 0.615 – 0.385*0.615 = 0.763 (76.3%), implying a null expectation of 9.92 units lost (C**f*_AB_(SI_A_, SI_B_)), or ~3.1 units remaining. The dominance effect *f*_AB_(SI_A_, SI_B_) is 8, as *f*_A_(SI_A_) = 8 > *f*_B_(SI_B_) = 5, implying a null expectation would of 5 units remaining (C - *f*_AB_(SI_A_, SI_B_)). Response units (quantities) may include any measures of abundance, biodiversity, or ecosystem functioning (including but not limited to population mortality).

**Additional null models**

Two other relevant null models include those of concentration addition (Loewe & Muischnek 1926),

*f*_AB_(SI_A_, SI_B_) = *f*_A_(SI_A_ + γSI_B_), with γ = SIx_A_/SIx_B_,

where *f*_A_(SI_Ax_) = *f*_B_(SI_Bx_) = half the effect size limit, and stressor addition (Liess et al. 2016),

*f*_AB_(SI_A_, SI_B_) = *F*_Strcap_(*F*_Strcap_^-1^(*f*_A_(SI_A_)) + *F*_Strcap_^-1^(*f*_B_(SI_B_))),

where *F*_strcap_ is the cumulative density function for the distribution of stress capacity and *F*_Strcap_^-1^ is the quantile function. These nulls require knowledge of stressor-response/effect relationships and sensitivity distributions (respectively) that are frequently not known in multiple stressor studies. The latter model (stressor addition) is also only defined for mortality effects.

Additionally, the compositional null provides community-level predictions from summing those of individual species but requires detailed population responses that are rarely presented. Here, the predicted response of species *i* to stressors A and B is

${x̂}_{i,\mathrm{AB}}= \left( 0,x_{i,0}+\sum_{j}^{s} (x_{i,j}-x_{i,0} \right)$,

where $x_{i,0}$ is the biomass of the unstressed community and $x_{i,j}$ is the biomass under stressor *j*. In the case of a two-stressor response, *S* is equal to 2 for stressors *j* = A and *j* = B (Thompson et al. 2018).

**References**

Liess, M., Foit, K., Knillmann, S., Sch€afer, R. B., Liess, H.-D. (2016). Predicting

the synergy of multiple stress effects. *Sci. Rep.*, 6, 32965.

Loewe, S., Muischnek, H. (1926). Über Kombinationswirkungen. *Naunyn-*

*Schmiedebergs Archiv für experimentelle Pathologie und Pharmakologie*, 114, 313–326.

Schäfer, R.B., Piggott, J.J. (2018). Advancing understanding and prediction in multiple stressor research through a mechanistic basis for null models. *Glob. Change Biol.*, 24, 1817–1826.

Thompson, P.L., MacLennan, M.M., Vinebrooke, R.D. (2018). An improved null model for assessing the net effects of multiple stressors on communities. *Glob. Change Biol.*, 24, 517–525.

**Supporting Information 2**

**Detailed results**

**Table 1.** Results of tests for residual heterogeneity (QE) and omnibus tests of moderators (QM) for weighted meta-analytic models.

| Response category | Null model | QE test of residual heterogeneity | QM test of moderators |
| --- | --- | --- | --- |
| Stressor pairs | Additive | QE(df = 273) = 922.7095, p-val < .0001 | QM(df = 7) = 25.6389, p-val = 0.0006 |
| Stressor pairs | Multiplicative | QE(df = 273) = 969.2561, p-val < .0001 | QM(df = 7) = 19.3078, p-val = 0.0073 |
| Stressor pairs | Dominance | QE(df = 273) = 897.2026, p-val < .0001 | QM(df = 7) = 24.7831, p-val = 0.0008 |
| Organisational level | Additive | QE(df = 285) = 978.0628, p-val < .0001 | QM(df = 2) = 3.0857,  p-val = 0.2138 |
| Organisational level | Multiplicative | QE(df = 285) = 993.7289, p-val < .0001 | QM(df = 2) = 2.3648,  p-val = 0.3065 |
| Organisational level | Dominance | QE(df = 285) = 989.3740, p-val < .0001 | QM(df = 2) = 2.8320,  p-val = 0.2427 |
| Asymmetric stressor | Additive | QE(df = 269) = 921.1329, p-val < .0001 | QM(df = 4) = 4.7191,  p-val = 0.3174 |
| Asymmetric stressor | Multiplicative | QE(df = 269) = 920.4537, p-val < .0001 | QM(df = 4) = 9.8817,  p-val = 0.0425 |
| Asymmetric stressor | Dominance | QE(df = 269) = 936.3636, p-val < .0001 | QM(df = 4) = 4.3732,  p-val = 0.3578 |
| Organism group | Additive | QE(df = 284) = 978.8024, p-val < .0001 | QM(df = 3) = 5.5567,  p-val = 0.1353 |
| Organism group | Multiplicative | QE(df = 284) = 990.6292, p-val < .0001 | QM(df = 3) = 2.4409,  p-val = 0.4861 |
| Organism group | Dominance | QE(df = 284) = 990.5583, p-val < .0001 | QM(df = 3) = 4.0140,  p-val = 0.2600 |
| Response metric | Additive | QE(df = 277) = 959.9710, p-val < .0001 | QM(df = 10) = 10.4884, p-val = 0.3987 |
| Response metric | Multiplicative | QE(df = 277) = 977.6223, p-val < .0001 | QM(df = 10) = 1.9143, p-val = 0.9970 |
| Response metric | Dominance | QE(df = 277) = 971.8042, p-val < .0001 | QM(df = 10) = 7.5066, p-val = 0.6769 |

*Notes:* Tests for residual heterogeneity indicate whether the variability in effects sizes left unexplained by moderators in the model is larger than expected given sampling variability (and covariances among sampling errors; i.e. significant tests suggest existence of other important moderators). The omnibus tests of moderators are Wald-type tests indicating whether model coefficients differ from zero (i.e. significant tests suggest that the moderators are important).

**Table 2*.*** Results for generalized linear regression models (Gamma error distribution and log-link function) investigating the relationship between accuracy of null model predictions (absolute effect sizes) and degree of stressor asymmetry depending on whether temperature warming or secondary stressors is the prevailing independent stressor.

| Model | Null | Intercept | Slope | df | AIC |
| --- | --- | --- | --- | --- | --- |
| Temperature prevailing | Additive | 0.0355040 | 0.0016830 | 34 | 99.46 |
|  | Multiplicative | -0.1333480 | 0.0018610 | 34 | 91.30 |
|  | Dominance | 0.0880250 | 0.0017840 | 34 | 81.25 |
| Secondary stressor prevailing | Additive | 0.4609415 | -0.0009462 | 52 | 137.20 |
|  | Multiplicative | 0.4238392 | 0.0008445 | 52 | 177.60 |
|  | Dominance | 0.3401800 | -0.0010620 | 52 | 119.10 |


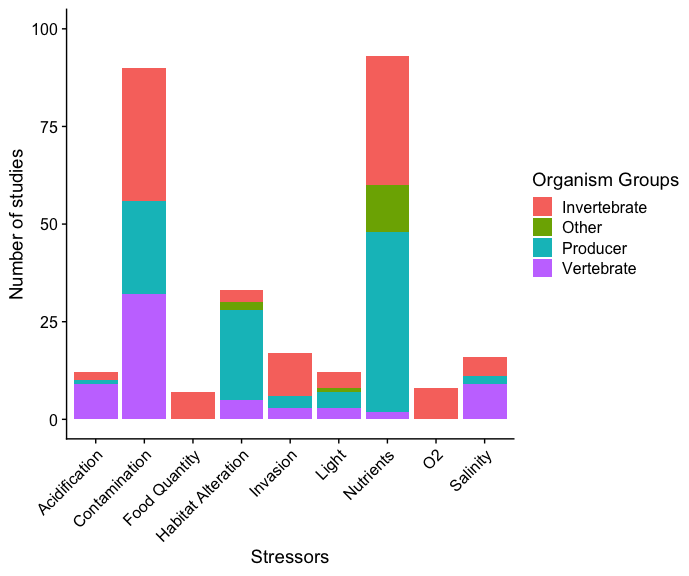


**SI Figure 1:** **Organism groups.** Number of studies in different organism groups across secondary stressor groups

**
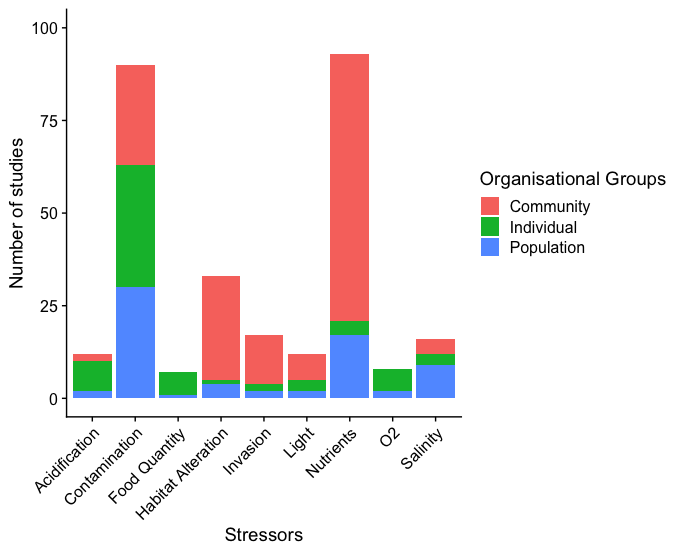
**

**SI Figure 2:** **Organisational groups.** Number of studies in different organisational groups across secondary stressor groups

**
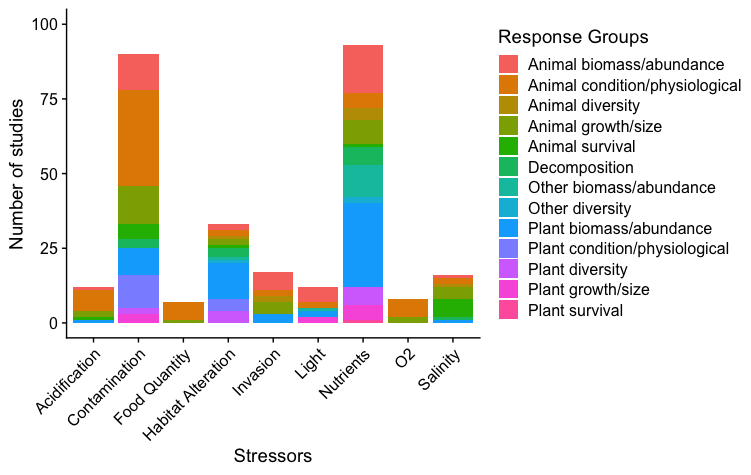
**

**SI Figure 3:** **Response groups.** Number of studies in different response metric groups across secondary stressor groups

**SI Figure 4:** **Organism group.** Frequency of responses best predicted by additive (blue), dominance (orange), and multiplicative (green) models (**a**) and their mean effect sizes (Hedges’ *d*) showing standardized differences between observed effects and those predicted for each null model (with 95% confidence intervals; **b**) across broad organism categories.

**SI Figure 5: Response metric group.** Frequency of responses best predicted by additive (blue), dominance (organce), and multiplicative (green) models (**a**) and their mean effect sizes (Hedges’ *d*) showing standardized differences between observed effects and those predicted for each null model (with 95% confidence intervals; **b**) across broad response metric categories.

**Supporting Information 3**

**Data reference list**

**Original data, from Jackson et al 2016**

Batista, D., Pascoal, C., & Cássio, F. (2012). Impacts of warming on aquatic decomposers along a gradient of cadmium stress. *Environmental Pollution*, *169*, 35–41.

Christensen, M. R., Graham, M. D., Vinebrooke, R. D., Findlay, D. L., Paterson, M. J., & Turner, M. A. (2006). Multiple anthropogenic stressors cause ecological surprises in boreal lakes. *Global Change Biology*, *12*(12), 2316–2322.

D’Cruz, L. M., Dockray, J. J., Morgan, I. J., & Wood, C. M. (1998). Physiological effects of sublethal acid exposure in juvenile rainbow trout on a limited or unlimited ration during a simulated global warming scenario. *Physiological Zoology*, *71*(4), 359–376.

De Senerpont Domis, L. N., Van de Waal, D. B., Helmsing, N. R., Van Donk, E., & Mooij, W. M. (2014). Community stoichiometry in a changing world: combined effects of warming and eutrophication on phytoplankton dynamics. *Ecology*, *95*(6), 1485–1495.

Dinh Van, K., Janssens, L., Debecker, S., De Jonge, M., Lambret, P., Nilsson-Örtman, V., Bervoets, L., & Stoks, R. (2013). Susceptibility to a metal under global warming is shaped by thermal adaptation along a latitudinal gradient. *Global Change Biology*, *19*(9), 2625–2633.

Dockray, J. J., Morgan, I. J., Reid, S. D., & Wood, C. M. (1998). Responses of juvenile rainbow trout, under food limitation, to chronic low pH and elevated summer temperatures, alone and in combination. *Journal of Fish Biology*, *52*(1), 62–82.

Domaizon, I., Lepère, C., Debroas, D., Bouvy, M., Ghiglione, J., Jacquet, S., Bettarel, Y., Bouvier, C., Torréton, J., Vidussi, F., Mostajir, B., Kirkham, A., LeFloc’h, E., Fouilland, E., Montanié, H., & Bouvier, T. (2012). Short-term responses of unicellular planktonic eukaryotes to increases in temperature and UVB radiation. *BMC Microbiology*, *12*(1), 202.

Doyle, S. A., Saros, J. E., & Williamson, C. E. (2005). Interactive effects of temperature and nutrient limitation on the response of alpine phytoplankton growth to ultraviolet radiation. *Limnology and Oceanography*, *50*(5), 1362–1367.

Greig, H. S., Kratina, P., Thompson, P. L., Palen, W. J., Richardson, J. S., & Shurin, J. B. (2012). Warming, eutrophication, and predator loss amplify subsidies between aquatic and terrestrial ecosystems. *Global Change Biology*, *18*(2), 504–514.

Heugens, E. H. W., Tokkie, L. T. B., Kraak, M. H. S., Hendriks, A. J., Van Straalen, N. M., & Admiraal, W. (2006). Population growth of Daphnia magna under multiple stress conditions: Joint effects of temperature, food, and cadmium. *Environmental Toxicology and Chemistry*, *25*(5), 1399–1407.

Holzapfel, A. M., & Vinebrooke, R. D. (2005). Environmental warming increases invasion potential of alpine lake communities by imported species. *Global Change Biology*, *11*(11), 2009–2015.

Jokinen, I. E., Salo, H. M., Markkula, E., Rikalainen, K., Arts, M. T., & Browman, H. I. (2011). Additive effects of enhanced ambient ultraviolet B radiation and increased temperature on immune function, growth and physiological condition of juvenile (parr) Atlantic Salmon, Salmo salar. *Fish & Shellfish Immunology*, *30*(1), 102–108.

Jun, Q., Pao, X., Haizhen, W., Ruiwei, L., & Hui, W. (2012). Combined effect of temperature, salinity and density on the growth and feed utilization of Nile tilapia juveniles (Oreochromis niloticus). *Aquaculture Research*, *43*(9), 1344–1356.

Kimberly, D. A., & Salice, C. J. (2014). Complex interactions between climate change and toxicants: evidence that temperature variability increases sensitivity to cadmium. *Ecotoxicology*, *23*(5), 809–817.

Knillmann, S., Stampfli, N. C., Noskov, Y. A., Beketov, M. A., & Liess, M. (2013). Elevated temperature prolongs long-term effects of a pesticide on Daphnia spp. due to altered competition in zooplankton communities. *Global Change Biology*, *19*(5), 1598–1609.

Kratina, P., Greig, H. S., Thompson, P. L., Carvalho-Pereira, T. S. A., & Shurin, J. B. (2012). Warming modifies trophic cascades and eutrophication in experimental freshwater communities. *Ecology*, *93*(6), 1421–1430.

Kuehne, L. M., Olden, J. D., & Duda, J. J. (2012). Costs of living for juvenile Chinook salmon (Oncorhynchus tshawytscha) in an increasingly warming and invaded world. *Canadian Journal of Fisheries and Aquatic Sciences*, *69*(10), 1621–1630.

Lahnsteiner, F., Haunschmid, R., & Mansour, N. (2011). Possible reasons for late summer brown trout (Salmo trutta Linnaeus 1758) mortality in Austrian prealpine river systems. *Journal of Applied Ichthyology*, *27*(1), 83–93.

Lapointe, D., Pierron, F., & Couture, P. (2011). Individual and combined effects of heat stress and aqueous or dietary copper exposure in fathead minnows (Pimephales promelas). *Aquatic Toxicology*, *104*(1–2), 80–85.

Linton, T. K., Reid, S. D., & Wood, C. M. (1998). The Metabolic Costs and Physiological Consequences to Juvenile Rainbow Trout of a Simulated Winter Warming Scenario in the Presence or Absence of Sublethal Ammonia. *Transactions of the American Fisheries Society*, *127*(4), 611–619.

Linton, T. K., Reid, S. D., & Wood, C. M. (1999). Effects of a Restricted Ration on the Growth and Energetics of Juvenile Rainbow Trout Exposed to a Summer of Simulated Warming and Sublethal Ammonia. *Transactions of the American Fisheries Society*, *128*(4), 758–763.

Loewen, C. J. G., & Vinebrooke, R. D. (2016). Regional diversity reverses the negative impacts of an alien predator on local species-poor communities. *Ecology*, *97*(10), 2740–2749.

McKee, D., Atkinson, D., Collings, S. E., Eaton, J. W., Gill, A. B., Harvey, I., Hatton, K., Heyes, T., Wilson, D., & Moss, B. (2003). Response of freshwater microcosm communities to nutrients, fish, and elevated temperature during winter and summer. *Limnology and Oceanography*, *48*(2), 707–722.

Moran, R., Harvey, I., Moss, B., Feuchtmayr, H., Hatton, K., Heyes, T., & Atkinson, D. (2010). Influence of simulated climate change and eutrophication on three-spined stickleback populations: A large scale mesocosm experiment. *Freshwater Biology*, *55*(2), 315–325.

Morgan, I. J., D’Cruz, L. M., Dockray, J. J., Linton, T. K., McDonald, D. G., & Wood, C. M. (1998). The effects of elevated winter temperature and sub-lethal pollutants (low pH, elevated ammonia) on protein turnover in the gill and liver of rainbow trout (Oncorhynchus mykiss). *Fish Physiology and Biochemistry*, *19*(4), 377–389.

Moss, B., Mckee, D., Atkinson, D., Collings, S. E., Eaton, J. W., Gill, A. B., Harvey, I., Hatton, K., Heyes, T., & Wilson, D. (2003). How important is climate? Effects of warming, nutrient addition and fish on phytoplankton in shallow lake microcosms. *Journal of Applied Ecology*, *40*(5), 782–792.

Muyssen, B. T. A., Messiaen, M., & Janssen, C. R. (2010). Combined cadmium and temperature acclimation in Daphnia magna: Physiological and sub-cellular effects. *Ecotoxicology and Environmental Safety*, *73*(5), 735–742.

Peuranen, S., Keinänen, M., Tigerstedt, C., & Vuorinen, P. J. (2003). Effects of temperature on the recovery of juvenile grayling (Thymallus thymallus) from exposure to Al+Fe. *Aquatic Toxicology*, *65*(1), 73–84.

Piggott, J. J., Lange, K., Townsend, C. R., & Matthaei, C. D. (2012). Multiple Stressors in Agricultural Streams: A Mesocosm Study of Interactions among Raised Water Temperature, Sediment Addition and Nutrient Enrichment. *PLoS ONE*, *7*(11), e49873.

Reese, C. D., & Harvey, B. C. (2002). Temperature-Dependent Interactions between Juvenile Steelhead and Sacramento Pikeminnow in Laboratory Streams. *Transactions of the American Fisheries Society*, *131*(4), 599–606.

Reid, S. D., Dockray, J. J., Linton, T. K., McDonald, D. G., & Wood, C. M. (1997). Effects of chronic environmental acidification and a summer global warming scenario: Protein synthesis in juvenile rainbow trout (Oncorhynchus mykiss). *Canadian Journal of Fisheries and Aquatic Sciences*, *54*(9), 2014–2024.

Rogell, B., Hofman, M., Eklund, M., Laurila, A., & HÖglund, J. (2009). The interaction of multiple environmental stressors affects adaptation to a novel habitat in the natterjack toad Bufo calamita. *Journal of Evolutionary Biology*, *22*(11), 2267–2277.

Seeland, A., Albrand, J., Oehlmann, J., & Müller, R. (2013). Life stage-specific effects of the fungicide pyrimethanil and temperature on the snail Physella acuta (Draparnaud, 1805) disclose the pitfalls for the aquatic risk assessment under global climate change. *Environmental Pollution*, *174*, 1–9.

Shrimpton, J. M., Zydlewski, J. D., & Heath, J. W. (2007). Effect of daily oscillation in temperature and increased suspended sediment on growth and smolting in juvenile chinook salmon, Oncorhynchus tshawytscha. *Aquaculture*, *273*(2–3), 269–276.

Shurin, J. B., Clasen, J. L., Greig, H. S., Kratina, P., & Thompson, P. L. (2012). Warming shifts top-down and bottom-up control of pond food web structure and function. *Philosophical Transactions of the Royal Society B: Biological Sciences*, *367*(1605), 3008–3017.

Tasmin, R., Shimasaki, Y., Tsuyama, M., Qiu, X., Khalil, F., Okino, N., Yamada, N., Fukuda, S., Kang, I. J., & Oshima, Y. (2014). Elevated water temperature reduces the acute toxicity of the widely used herbicide diuron to a green alga, Pseudokirchneriella subcapitata. *Environmental Science and Pollution Research*, *21*(2), 1064–1070.

Thompson, P. L., & Shurin, J. B. (2012). Regional zooplankton biodiversity provides limited buffering of pond ecosystems against climate change. *Journal of Animal Ecology*, *81*(1), 251–259.

Thompson, P. L., St-Jacques, M.-C., & Vinebrooke, R. D. (2008). Impacts of Climate Warming and Nitrogen Deposition on Alpine Plankton in Lake and Pond Habitats: an In Vitro Experiment. *Arctic, Antarctic, and Alpine Research*, *40*(1), 192–198.

Vidussi, F., Mostajir, B., Fouilland, E., Le Floc’H, E., Nouguier, J., Roques, C., Got, P., Thibault-Botha, D., Bouvier, T., & Troussellier, M. (2011). Effects of experimental warming and increased ultraviolet B radiation on the Mediterranean plankton food web. *Limnology and Oceanography*, *56*(1), 206–218.

Wagner, E. J., Bosakowski, T., & Intelmann, S. (1997). Combined Effects of Temperature and High pH on Mortality and the Stress Response of Rainbow Trout after Stocking. *Transactions of the American Fisheries Society*, *126*(6), 985–998.

Weisse, T., Laufenstein, N., & Weithoff, G. (2013). Multiple environmental stressors confine the ecological niche of the rotifer Cephalodella acidophila. *Freshwater Biology*, *58*(5), 1008–1015.

**Additional data**

Barbosa, M., Inocentes, N., Soares, A. M. V. M., & Oliveira, M. (2017). Synergy effects of fluoxetine and variability in temperature lead to proportionally greater fitness costs in Daphnia: A multigenerational test. *Aquatic Toxicology*, *193*, 268–275.

Correa-Araneda, F., Basaguren, A., Abdala-Díaz, R. T., Tonin, A. M., & Boyero, L. (2017). Resource-allocation tradeoffs in caddisflies facing multiple stressors. *Ecology and Evolution*, *7*(14), 5103–5110.

Cuenca Cambronero, M., Marshall, H., De Meester, L., Davidson, T. A., Beckerman, A. P., & Orsini, L. (2018). Predictability of the impact of multiple stressors on the keystone species Daphnia. *Scientific Reports*, *8*(1), 17572.

Dinh, K. V., Janssens, L., & Stoks, R. (2016). Exposure to a heat wave under food limitation makes an agricultural insecticide lethal: a mechanistic laboratory experiment. *Global Change Biology*, *22*(10), 3361–3372.

Ferreira-Rodríguez, N., & Pardo, I. (2017). The interactive effects of temperature, trophic status, and the presence of an exotic clam on the performance of a native freshwater mussel. *Hydrobiologia*, *797*(1), 171–182.

Gandar, A., Jean, S., Canal, J., Marty-Gasset, N., Gilbert, F., & Laffaille, P. (2016). Multistress effects on goldfish (Carassius auratus) behavior and metabolism. *Environmental Science and Pollution Research*, *23*(4), 3184–3194.

Hani, Y. M. I., Turies, C., Palluel, O., Delahaut, L., Bado-Nilles, A., Geffard, A., Dedourge-Geffard, O., & Porcher, J.-M. (2019). Effects of a chronic exposure to different water temperatures and/or to an environmental cadmium concentration on the reproduction of the threespine stickleback (Gasterosteus aculeatus). *Ecotoxicology and Environmental Safety*, *174*, 48–57.

Hasenbein, S., Poynton, H., & Connon, R. E. (2018). Contaminant exposure effects in a changing climate: how multiple stressors can multiply exposure effects in the amphipod Hyalella azteca. *Ecotoxicology*, *27*(7), 845–859.

Henry, Y., Piscart, C., Charles, S., & Colinet, H. (2017). Combined effect of temperature and ammonia on molecular response and survival of the freshwater crustacean Gammarus pulex. *Ecotoxicology and Environmental Safety*, *137*, 42–48.

Hoefnagel, K. N., & Verberk, W. C. E. P. (2017). Long-term and acute effects of temperature and oxygen on metabolism, food intake, growth and heat tolerance in a freshwater gastropod. *Journal of Thermal Biology*, *68*, 27–38.

Hopkins, G. R., French, S. S., & Brodie, E. D. (2017). Interacting stressors and the potential for adaptation in a changing world: responses of populations and individuals. *Royal Society Open Science*, *4*(6), 161057.

Lambert, A. S., Dabrin, A., Foulquier, A., Morin, S., Rosy, C., Coquery, M., & Pesce, S. (2017). Influence of temperature in pollution-induced community tolerance approaches used to assess effects of copper on freshwater phototrophic periphyton. *Science of The Total Environment*, *607*–*608*, 1018–1025.

Lambert, A. S., Dabrin, A., Morin, S., Gahou, J., Foulquier, A., Coquery, M., & Pesce, S. (2016). Temperature modulates phototrophic periphyton response to chronic copper exposure. *Environmental Pollution*, *208*, 821–829.

Lopez, L. K., Davis, A. R., & Wong, M. Y. L. (2018). Behavioral interactions under multiple stressors: temperature and salinity mediate aggression between an invasive and a native fish. *Biological Invasions*, *20*(2), 487–499.

Loureiro, C., Cuco, A. P., Claro, M. T., Santos, J. I., Pedrosa, M. A., Gonçalves, F., & Castro, B. B. (2015). Progressive acclimation alters interaction between salinity and temperature in experimental Daphnia populations. *Chemosphere*, *139*, 126–132.

MacLennan, M. M., & Vinebrooke, R. D. (2016). Effects of non-native trout, higher temperatures and regional biodiversity on zooplankton communities of alpine lakes. *Hydrobiologia*, *770*(1), 193–208.

Mari, L., Garaud, L., Evanno, G., & Lasne, E. (2016). Higher temperature exacerbates the impact of sediments on embryo performances in a salmonid. *Biology Letters*, *12*(12).

Morin, S., Lambert, A. S., Rodriguez, E. P., Dabrin, A., Coquery, M., & Pesce, S. (2017). Changes in copper toxicity towards diatom communities with experimental warming. *Journal of Hazardous Materials*, *334*, 223–232.

O’Mara, K. M., & Wong, M. Y. L. (2016). Body size mediated effects of multiple abiotic stressors on the growth and social behaviour of an estuarine fish, Australian Bass (Macquaria novemaculeata). *Environmental Biology of Fishes*, *99*(1), 95–104.

Olsen, S., Cao, Y., Florencia Gutierrez, M., Brucet, S., Landkildehus, F., Lauridsen, T. L., Davidson, T. A., Søndergaard, M., Jeppesen, E., & Risgaard-Petersen, N. (2017). Effect of a nitrogen pulse on ecosystem N processing at different temperatures: A mesocosm experiment with ^15^ NO _3_ ^−^ addition. *Freshwater Biology*, *62*(7), 1232–1243.

Pesce, S., Lambert, A.-S., Morin, S., Foulquier, A., Coquery, M., & Dabrin, A. (2018). Experimental Warming Differentially Influences the Vulnerability of Phototrophic and Heterotrophic Periphytic Communities to Copper Toxicity. *Frontiers in Microbiology*, *9*, 1424.

Piggott, J. J., Niyogi, D. K., Townsend, C. R., & Matthaei, C. D. (2015). Multiple stressors and stream ecosystem functioning: climate warming and agricultural stressors interact to affect processing of organic matter. *Journal of Applied Ecology*, *52*(5), 1126–1134.

Piggott, J. J., Salis, R. K., Lear, G., Townsend, C. R., & Matthaei, C. D. (2015). Climate warming and agricultural stressors interact to determine stream periphyton community composition. *Global Change Biology*, *21*(1), 206–222.

Ren, L., He, D., Chen, Z., Jeppesen, E., Lauridsen, T. L., Søndergaard, M., Liu, Z., & Wu, Q. L. (2017). Warming and nutrient enrichment in combination increase stochasticity and beta diversity of bacterioplankton assemblages across freshwater mesocosms. *ISME Journal*, *11*(3), 613–625.

Romero, F., Sabater, S., Timoner, X., & Acuña, V. (2018). Multistressor effects on river biofilms under global change conditions. *Science of The Total Environment*, *627*, 1–10.

Salo, T., Räsänen, K., Stamm, C., Burdon, F. J., & Seppälä, O. (2018). Simultaneous exposure to a pulsed and a prolonged anthropogenic stressor can alter consumer multifunctionality. *Oikos*, *127*(10), 1437–1448.

Šorf, M., Davidson, T. A., Brucet, S., Menezes, R. F., Søndergaard, M., Lauridsen, T. L., Landkildehus, F., Liboriussen, L., & Jeppesen, E. (2015). Zooplankton response to climate warming: a mesocosm experiment at contrasting temperatures and nutrient levels. *Hydrobiologia*, *742*(1), 185–203.

Velthuis, M., van Deelen, E., van Donk, E., Zhang, P., & Bakker, E. S. (2017). Impact of Temperature and Nutrients on Carbon: Nutrient Tissue Stoichiometry of Submerged Aquatic Plants: An Experiment and Meta-Analysis. *Frontiers in Plant Science*, *8*, 655.

Wen, B., Zhang, N., Jin, S. R., Chen, Z. Z., Gao, J. Z., Liu, Y., Liu, H. P., & Xu, Z. (2018). Microplastics have a more profound impact than elevated temperatures on the predatory performance, digestion and energy metabolism of an Amazonian cichlid. *Aquatic Toxicology*, *195*, 67–76.

Yong, W. K., Sim, K. S., Poong, S. W., Wei, D., Phang, S. M., & Lim, P. E. (2018). Interactive effects of temperature and copper toxicity on photosynthetic efficiency and metabolic plasticity in Scenedesmus quadricauda (Chlorophyceae). *Journal of Applied Phycology*, *30*(6), 3029–3041.
